# Supplementary material for: Adverse Effects of Oral Cannabidiol: An Updated Systematic Review of Randomized Controlled Trials (2020–2022)
Source: Pharmaceutics. 2022 Nov 25;14(12):2598. doi: 10.3390/pharmaceutics14122598 (PMC9782576; doi:10.3390/pharmaceutics14122598)
Supplement: Supplementary file 1 [file pharmaceutics-14-02598-s001.zip › pharmaceutics-2006193-supplementary.pdf]

Supplemental Online Content

Souza et. al.; Adverse Effects of Oral Cannabidiol: An Updated Systematic Review of Randomized Controlled Trials

Methodological adaptations compared to the previous review

Inclusion criteria

Present review: Oral purified Cannabidiol formulations (≥ 98% CBD).

Previous review: Pure CBD or standardized CBD extracts with <3% THC.

Databases

Present review: EMBASE, MEDLINE/PubMed, and Web of Science.

Previous review: PubMed.

Terms

Present review: "(cannabidiol OR CBD) AND (randomized clinical trial OR double-blind OR placebo-controlled)".

Previous review: “(cannabidiol) AND (randomized clinical trial OR double-blind OR placebo controlled)”.

Table S1. Quality Assessment of Controlled Intervention Studies

Quality Assessment of Controlled Intervention Studies			
**Criteria**	**Yes**	**No**	**Other (CD, NR, NA)***
1. Was the study described as randomized, a randomized trial, a randomized clinical trial, or an RCT?			
2. Was the method of randomization adequate (i.e., use of randomly generated assignment)?			
3. Was the treatment allocation concealed (so that assignments could not be predicted)?			
4. Were study participants and providers blinded to treatment group assignment?			
5. Were the people assessing the outcomes blinded to the participants' group assignments?			
6. Were the groups similar at baseline on important characteristics that could affect outcomes (e.g., demographics, risk factors, co-morbid conditions)?			
7. Was the overall drop-out rate from the study at endpoint 20% or lower of the number allocated to treatment?			
8. Was the differential drop-out rate (between treatment groups) at endpoint 15 percentage points or lower?			
9. Was there high adherence to the intervention protocols for each treatment group?			
10. Were other interventions avoided or similar in the groups (e.g., similar background treatments)?			
11. Were outcomes assessed using valid and reliable measures, implemented consistently across all study participants?			
12. Did the authors report that the sample size was sufficiently large to be able to detect a difference in the main outcome between groups with at least 80% power?			
13. Were outcomes reported or subgroups analyzed prespecified (i.e., identified before analyses were conducted)?			
14. Were all randomized participants analyzed in the group to which they were originally assigned, i.e., did they use an intention-to-treat analysis?			

The quality of included studies was assessed using the National Institutes of Health (NIH) Quality Assessment of Controlled Intervention Studies (https://www.nhlbi.nih.gov/health-pro/guidelines/in-develop/cardiovascular-risk-reduction/tools/cohort).

Total Score: Number of yes; **CD,** cannot be determined; **NA,** not applicable; **NR,** not reported.
Quality Rating: Poor <50%, Fair 50-75%, Good ≥75%.

Quality Assessment of Controlled Intervention Studies toll - Items reported in each study

Appiah-Kusi et al., 2020 [10]			
Criteria	Yes	No	(CD, NR, NA)
1	■		
2			NR
3			NR
4	■		
5	■		
6	■		
7			NR
8	■		
9	■		
10	■		
11	■		
12		■	
13	■		
14		■	

Crippa et al., 2021 [17]			
Criteria	Yes	No	(CD, NR, NA)
1	■		
2	■		
3	■		
4	■		
5	■		
6	■		
7	■		
8	■		
9	■		
10	■		
11	■		
12	■		
13	■		
14	■		

Atieh et al., 2022 [21]			
Criteria	Yes	No	(CD, NR, NA)
1	■		
2	■		
3	■		
4	■		
5	■		
6		■	
7		■	
8	■		
9	■		
10		■	
11	■		
12	■		
13	■		
14		■	

De Almeida et al., 2021 [18]			
Criteria	Yes	No	(CD, NR, NA)
1	■		
2	■		
3			NR
4	■		
5	■		
6	■		
7	■		
8	■		
9	■		
10		■	
11	■		
12	■		
13	■		
14		■	

Ben-Menachem et al., 2020 [11]			
Criteria	Yes	No	(CD, NR, NA)
1	■		
2			NR
3			NR
4	■		
5	■		
6	■		
7	■		
8	■		
9	■		
10	■		
11	■		
12		■	
13	■		
14			

Efron et al., 2020 [12]			
Criteria	Yes	No	(CD, NR, NA)
1	■		
2			NR
3	■		
4	■		
5	■		
6		■	
7	■		
8	■		
9	■		
10		■	
11	■		
12		■	
13	■		
14	■		

Freeman et al., 2020 [13]			
Criteria	Yes	No	(CD, NR, NA)
1			
2			
3			
4			
5			
6			
7			
8			
9			
10			
11			
12			
13			
14			

Mongeau-Pérusse et al., 2021 [20]			
Criteria	Yes	No	(CD, NR, NA)
1			
2			
3			
4			
5			
6			
7			
8			
9			
10			
11			
12			
13			
14			

Leweke et al., 2021 [19]			
Criteria	Yes	No	(CD, NR, NA)
1			
2			NR
3			NR
4			
5			
6			
7			
8			
9			
10			
11			
12			
13			
14			

Thiele et al., 2020 [15]			
Criteria	Yes	No	(CD, NR, NA)
1			
2			NR
3			NR
4			
5			
6			
7			
8			
9			
10			
11			
12			
13			
14			

Meneses-Gaya et al., 2020 [14]			
Criteria	Yes	No	(CD, NR, NA)
1			
2			NR
3			NR
4			
5			
6			
7			
8			
9			
10			
11			
12			NR
13			
14			

VanLandingham et al., 2020 [16]			
Criteria	Yes	No	(CD, NR, NA)
1			
2			NR
3			NR
4			
5			
6			
7			
8			
9			
10			
11			
12			
13			
14			

Table S2. Serious Adverse Events in ≥1 Patient

Adverse Effects, No.	Placebo	CBD20	CBD25	CBD50
Increased ALT/AST	-	1	9	19
Seizure	1	1	3	2
Rash	-	1	2	2
Vomiting	-	-	2	-
Gastroenteritis	-	-	2	1
Pneumonia	1	-	2	-
Abdominal pain	-	-	-	1
Acute respiratory failure	-	-	1	-
Angioedema	-	-	-	1
Blood bilirubin increased	-	-	1	-
Dehydration	-	-	-	1
Diarrhea	-	-	-	1
Electrolyte imbalance	-	-	1	-
Fatigue		-	1	-
Hepatitis	1	-	-	-
Hypophagia	-	-	1	-
Laceration	-	-	-	1
Liver injury	-	-	1	-
Malaise	-	-	1	-
Nausea	-	-	1	-
Otitis media acute	-	-	1	-
Toxicity to various agents	-	-	-	1
Type IV hypersensitivity reaction	-	-	1	-

CBD20 20mg/kg/d; *CBD25* 25mg/kg/d; *CBD50* 50mg/kg/d.
